# Immunomodulatory Effects of Clinically Used Fat Emulsion to Promote Angiogenesis and Osteogenesis for Bone Repair

**DOI:** 10.3390/ma19071290

**Published:** 2026-03-24

**Authors:** Luyao Cheng, Zetao Wang, Yujie Liu, Yuyang Zhang, Yu Gao, Tianyi Zhou, Yuxiao Lai, Wei Zhang

**Affiliations:** 1 School of Biomedical Engineering, Southern University of Science and Technology, Shenzhen 518055, China; 2 College of Medical Engineering, Shenzhen University of Advanced Technology, Shenzhen 518055, China; 3 Centre for Translational Medicine Research & Development, Shenzhen Institutes of Advanced Technology, Chinese Academy of Sciences, Shenzhen 518055, Chinayu.gao@siat.ac.cn (Y.G.); 4 School of Biomedical Sciences and Engineering, South China University of Technology, Guangzhou International Campus, Guangzhou 511442, China; 5 National Innovation Center for Advanced Medical Devices, Shenzhen 518131, China; 6 Department of Bone & Joint Surgery, Peking University Shenzhen Hospital, Shenzhen 518036, China

**Keywords:** fat emulsion, immunomodulation, angiogenesis, osteogenic differentiation

## Abstract

Bone defects have become a leading cause of disability and mortality. The pro-inflammatory state and impaired vascularization are major factors hindering bone defect repair. However, current bone regeneration materials lack the ability to regulate the osteoimmune microenvironment and promote vascularized bone regeneration. In this study, we employed clinically used fat emulsion (FE), which is intravenously administered to provide nutrition and energy for patients, to investigate the effect of immunomodulation on promoting angiogenesis and osteogenesis. Results from RT-qPCR analysis and immunofluorescence staining demonstrated that FE exhibited potent anti-inflammatory effects by reducing the expression of the pro-inflammatory marker inducible nitric oxide synthase (iNOS) and upregulating the expression of the anti-inflammatory marker transforming growth factor-beta (TGF-β). Endothelial tube formation and scratch assays demonstrated that FE promoted angiogenesis and cell migration by releasing vascular endothelial growth factor (VEGF) within the inflammatory microenvironment. Alkaline phosphatase (ALP) and alizarin red S (ARS) staining revealed that FE facilitated ALP activity and calcium nodule formation by releasing bone morphogenetic protein-2 (BMP-2) within the inflammatory microenvironment. These findings may prove promising and cost-effective for the clinical treatment of bone defects.

## 1. Introduction

Bone defects present a common clinical challenge, and their repair requires not only new bone formation but also a supportive immune environment and adequate blood supply [1,2,3,4,5,6,7,8,9]. Following bone defect formation, local blood supply disruption frequently occurs, leading to insufficient oxygen and nutrient delivery that severely impedes the bone repair process [5,6,10,11]. Concurrently, trauma triggers persistent inflammatory responses characterized by massive infiltration of inflammatory cells and cytokine release, further impairing new blood vessel formation and creating a vicious cycle that impedes recovery [11,12,13,14,15]. In recent years, numerous studies have highlighted the role of immune modulation in bone repair [16,17,18]. However, most biomaterials face challenges in clinical translation. Therefore, developing materials with clinical translation potential that can regulate immune responses to promote bone regeneration is of significant importance.

Local vascular injury induces ischemia and hypoxia, leading to elevated hypoxia inducible factor 1α (HIF-1α) and vascular endothelial growth factor (VEGF) expression [19,20]. Meanwhile, in the early stages of bone defects, M1 type macrophages release inflammatory mediators, including inducible nitric oxide synthase (iNOS) and interleukin-6 (IL-6), triggering acute inflammatory responses [21,22], and the excessive inflammation disrupts VEGF signaling, impairing angiogenesis and resulting in immature vascular function [23]. Therefore, modulating the early immune response, promoting macrophage polarization toward the M2 type to restore vascularization capacity are critical to breaking this cycle and achieving bone regeneration [24,25].

As a mature parenteral nutrition formulation, FE has expanded its clinical application beyond traditional nutritional support into multiple therapeutic domains. In perioperative cancer management, Tong et al.’s study of 88 patients undergoing radical resection for hilar cholangiocarcinoma demonstrated that, compared to conventional medium/long-chain fat emulsions, multi-oil fat emulsions significantly improved postoperative nutritional status while reducing levels of ALT, AST, total bilirubin, CRP, TNF-α, and IL-6. The complication rate decreased from 20.46% to 4.54%, confirming its dual anti-inflammatory and hepatoprotective effects [26]. In the field of neonatal nutritional support, Yao et al.’s study of 60 preterm infants revealed that the observation group receiving SMOF fat emulsion demonstrated superior immune function and clinical outcomes compared to the control group, alongside a lower incidence of complications [27]. In the field of neonatal nutritional support, Yao et al.’s study of 60 preterm infants revealed that the observation group receiving SMOF fat emulsion demonstrated superior immune function and clinical outcomes compared to the control group, alongside a lower incidence of complications [28].

Polyunsaturated fatty acids play a crucial role in inflammation regulation [29]. Specifically, ω-3 fatty acids actively promote inflammation resolution through distinct mechanisms by competitively inhibiting pro-inflammatory mediators and metabolizing into specific anti-inflammatory mediators such as resolvins and protectins [30,31]. As a precursor to pro-inflammatory signaling molecules like prostaglandins and leukotrienes, the ω-6 fatty acid arachidonic acid is indispensable for initiating immune responses [32,33]. Although oleic acid (ω-9 fatty acid) is non-essential, it indirectly exerts anti-inflammatory effects by enhancing cell membrane stability and partially replacing the uptake of pro-inflammatory lipids [34]. Fat emulsion (FE), emulsified fats rich in polyunsaturated fatty acids (including ω-3, ω-6, and ω-9), has long been developed as a dietary supplement for clinical use to provide nutrition and energy for patients through intravenous (*i.v.*) injection [35].

**Scheme 1:**
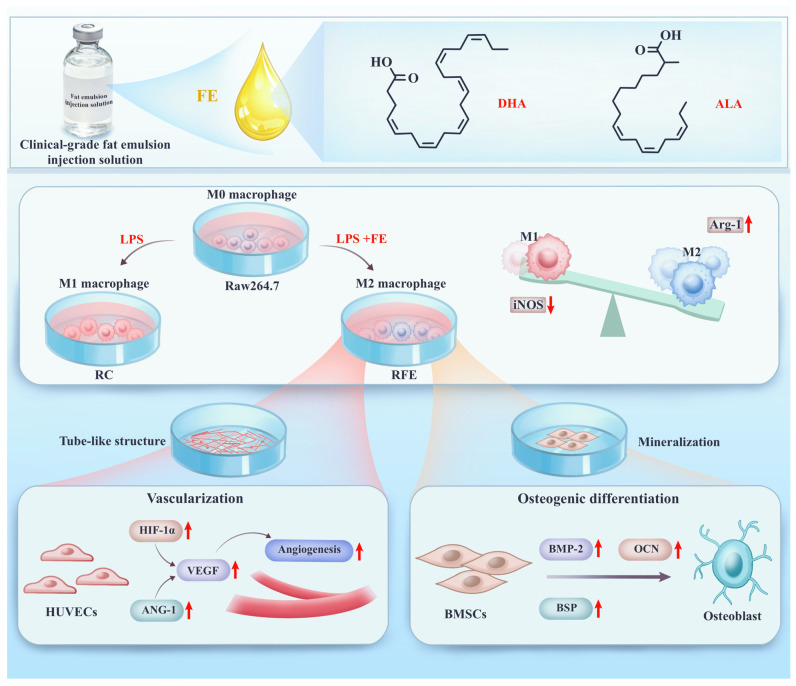
Schematic diagram of FE for promoting angiogenesis and osteogenesis through immunomodulation to accelerate bone regeneration.

In light of these bioactive properties, FE emerges as a promising candidate for simultaneously suppressing inflammation while promoting vascular remodeling and bone regeneration. In this study, the potential of FE to promote angiogenesis and osteogenesis through immunomodulation was investigated in vitro. FE mediated potent anti-inflammatory activity by significantly downregulating the expression of the pro-inflammatory marker iNOS and upregulating the expression of the anti-inflammatory cytokine transforming growth factor-beta (TGF-β). Furthermore, within an inflammatory microenvironment, FE was shown to promote angiogenesis and cell migration by upregulating the expression of VEGF, and to facilitate alkaline phosphatase (ALP) activity and calcium nodule formation by upregulating the expression of bone morphogenetic protein-2 (BMP-2). These findings suggest FE represents a promising therapeutic strategy for clinical bone defect repair (Scheme 1).

## 2. Materials and Methods

### 2.1. Materials

Medium/long-chain FE injection was procured from Guangdong Jiabo Pharmaceutical Co., Ltd. (Guangzhou, China). The human 22-carbon hexaenoic acid ELISA kit was purchased from Tianjin Yue Teng Biotechnology Co., Ltd. (Tianjin, China) Dulbecco’s Modified Eagle Medium (DMEM), α-Minimum Essential Medium (α-MEM), fetal bovine serum (FBS), and penicillin/streptomycin (P/S) were all purchased from Gibco (New York, NY, USA). The Cell Counting Kit-8 (CCK-8) was procured from MIKX (Shenzhen, China). Lipopolysaccharide (LPS) was obtained from Sigma (St. Louis, MO, USA). The Total RNA Extraction Kit was sourced from Novazene (Nanjing, China). The Real-time Quantitative Reverse Transcription Polymerase Chain Reaction (RT-qPCR) Kit was purchased from Takara (Dalian, China). The Calcein-AM/PI Cell Viability and Cytotoxicity Assay Kit was procured from Beyotime Biotechnology (Shanghai, China). μ-Slide 15-well 3D culture plates and Matrigel basement membrane matrix were both purchased from ibidi (Grafenberg, Germany). Formaldehyde tissue fixative was sourced from TargetMol (Shanghai, China). The BCIP/NBT alkaline phosphatase color development kit and Alizarin Red S (ARS) staining kit were both purchased from Beyotime Biotechnology (Shanghai, China).

### 2.2. Characterization of FE

The morphology and structure of FE were characterized using transmission electron microscopy (TEM, JEOL, JEM-200CX, Tokyo, Japan). The surface potential and particle size of FE were determined via nanoparticle size and zeta potential analysis (Zetasizer Nano-ZS, Malvern Instruments Ltd., Malvern, UK). The DHA content in FE was assessed using a human docosahexaenoic acid ELISA kit.

### 2.3. Cell Culture

Abelson murine leukemia virus-induced tumor 264.7 (Raw 264.7), Human Umbilical Vein Endothelial Cells (HUVECs) and Human Bone Marrow-Derived Mesenchymal Stem Cells (hBMSCs) were all obtained from the American Type Culture Collection (ATCC, Manassas, VA, USA).

Raw 264.7 cells and HUVECs were all cultured using DMEM. During culture, 10% FBS and 1% P/S (by volume) were added. Cells were maintained at 37 °C in a 5% CO_2_ incubator, with medium changes performed every two days.

hBMSCs were cultured using α-MEM medium supplemented with 10% FBS and 1% P/S (*v*/*v*). Cells were maintained at 37 °C in a 5% CO_2_ incubator, with medium changes performed every two days.

### 2.4. Biocompatibility Assessment

To evaluate the biocompatibility of FE, three cell types were seeded at appropriate densities in 96-well plates. To each well, 100 µL of complete medium containing different concentrations of FE was added, and cells were cultured for 0, 1, 2, and 3 days. Cell proliferation capacity was assessed using the CCK-8 assay kit, with optical density (OD) values measured at 450 nm wavelength using a microplate reader (SpectraMax M4, Molecular Devices, San Jose, CA, USA). Cell viability and toxicity were assessed using the Calcein-AM/PI staining kit. Cells were seeded at appropriate densities in 12-well plates. Each well was supplemented with 1 mL of complete medium containing different concentrations of FE. After 1 day of culture, cells were stained with a 1000-fold diluted staining solution for 15 min. Imaging was performed using a fluorescence live-cell imaging system (Olympus IX73P1F, Tokyo, Japan), and live/dead cell states were observed and recorded.

### 2.5. RT-qPCR Analysis

Real-time quantitative PCR was employed to analyze mRNA expression levels of pro-inflammatory, anti-inflammatory, osteogenic, and angiogenesis-related genes. Following FE treatment, cells were harvested at different time points. RNA was extracted using a total RNA extraction kit and subsequently reverse transcribed into cDNA. A 10 µL reaction mixture containing TB Green, cDNA, and specific primers (Table 1) was prepared. Target genes were amplified using the LightCycler 96 system (Roche, Basel, Switzerland). Glyceraldehyde-3-phosphate dehydrogenase (GAPDH) was employed as an internal control to normalize the mRNA expression levels of different target genes. We calculated the relative expression levels of genes using the 2^ΔΔCT^ method.

### 2.6. Immunofluorescence Staining

Immunofluorescence staining was employed to analyze protein levels associated with pro-inflammatory and anti-inflammatory processes. Cells were seeded in confocal culture dishes and treated with complete medium containing FE for one day before being fixed with formalin tissue fixative for 30 min. Subsequently, staining was performed using anti-iNOS and anti-Arg-1 antibodies, and images were acquired via a laser scanning confocal microscope (LSM900, ZEISS, Oberkochen, Germany).

### 2.7. Preparation of Immunomodulatory Conditioned Medium

**Figure 1:**
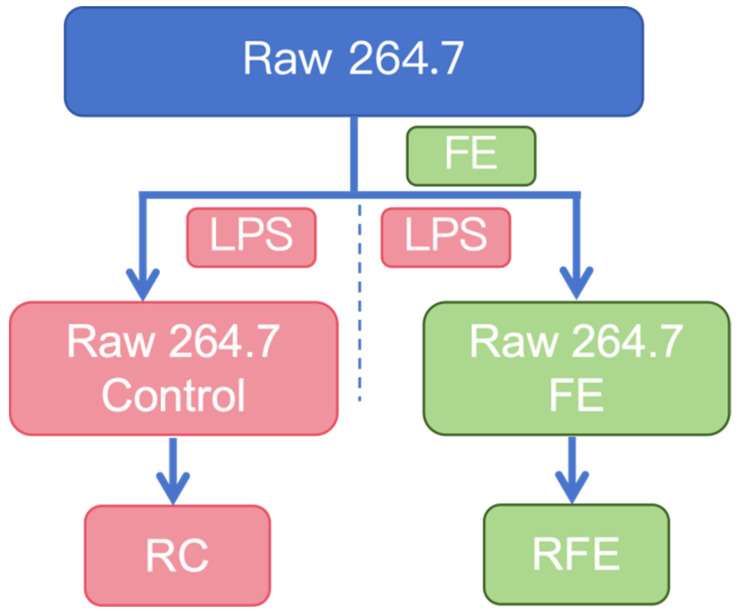
Preparation of immunomodulatory culture medium.

To simulate the effects of the inflammatory microenvironment on angiogenesis and osteogenesis in vitro, conditioned culture medium of Raw 264.7 cells was prepared. Raw 264.7 cells were cultured with LPS (1 μM), or LPS (1 μM) plus FE (200 μg/mL), for 1 day. Cell debris was removed from the cell culture supernatant collected by centrifugation to prepare Raw 264.7 conditioned medium (RC) and Raw 264.7 conditioned medium containing FE (RFE) (Figure 1). The conditioned medium prepared as described above was mixed with osteogenic induction solution in a 1:1 ratio to obtain osteogenic induction medium. Concurrently, the conditioned medium was mixed with DMEM complete medium in a 1:1 ratio to obtain angiogenic induction medium. These two media were subsequently employed for studies on osteogenic differentiation and angiogenesis under distinct immunological conditions.

### 2.8. Transwell Assay

The migration capacity of HUVECs was evaluated using the Transwell assay. Cells were resuspended in serum-free medium and seeded at an appropriate density into the upper chamber of Transwell plates. The lower chamber was filled with serum-free conditioned medium. After 12 h of incubation, cells were fixed with tissue fixative and stained with crystal violet. Non-migrated cells on the inner surface of the upper chamber membrane were carefully removed using a cotton swab. The number of cells migrating to the lower surface of the membrane was quantified by microscopic observation and counting to assess cell migration capacity.

### 2.9. Wound Healing Assay

Cell migration capacity was assessed using a wound healing assay. HUVECs were seeded at an appropriate cell density into ibidi four-well culture inserts within a 12-well plate and culture until 100% confluence is achieved. Subsequently, the four-well inserts were removed, and the complete medium was replaced with serum-free conditioned medium. Cell migration capacity was determined by measuring scratch width. Scratch width was recorded under a microscope at 0, 24, and 48 h. ImageJ software (ImageJ 1.54g, National Institutes of Health, Bethesda, MD, USA) was then used to measure scratch width at different time points and calculate the cell migration rate.

### 2.10. Tube Formation Assay

Collagen matrix solution was mixed with DMEM at a 1:1 ratio. After adding 15 µL of the mixture to ibidi culture slides at 4 °C, the slides were incubated at 37 °C for 30 min. Subsequently, 50 µL of cell suspension was seeded onto the gel surface at a density of 1.2 × 10^5^ cells per well. After 6 h, cells were stained using Calcein dye (diluted 1:1000). Following staining, tubular structures formed and were imaged under a microscope. The total length and number of nodes were analyzed using ImageJ.

### 2.11. ALP Staining

hBMSCs were seeded at an appropriate density into 12-well plates. After reaching 80–90% confluence, the complete medium was removed and osteogenic induction medium from different groups was added to induce osteogenesis. After 7 and 14 days of induction, cells were fixed with tissue fixative and stained with the BCIP/NBT alkaline phosphatase staining kit for 30 min. Results were ultimately observed under a microscope.

### 2.12. ARS Staining

The osteogenic mineralization capacity of hBMSCs was detected using the ARS staining method. hBMSCs were seeded at an appropriate cell density in 12-well plates and cultured until 80–90% confluence. The complete culture medium was then aspirated, and osteogenic induction medium from different groups was added for osteogenic induction. After 14 days of osteogenic induction, cells were fixed with tissue fixative, followed by ARS staining for 5 min. After two washes with ddH_2_O, samples were observed under a microscope. ARS was dissolved using a 10% hexadecylpyridinium chloride (CPC) solution, and absorbance was measured at 562 nm.

### 2.13. Statistical Analysis

Statistical analysis was performed using GraphPad Prism 9 software (San Diego, CA, USA). Each group comprised at least three samples, with all group data expressed as mean ± standard deviation (SD). Comparisons between multiple groups were conducted using one-way analysis of variance (ANOVA). * *p* < 0.05 was considered statistically significant.

## 3. Results and Discussion

### 3.1. Characteristics and Analysis of FE

**Figure 2:**
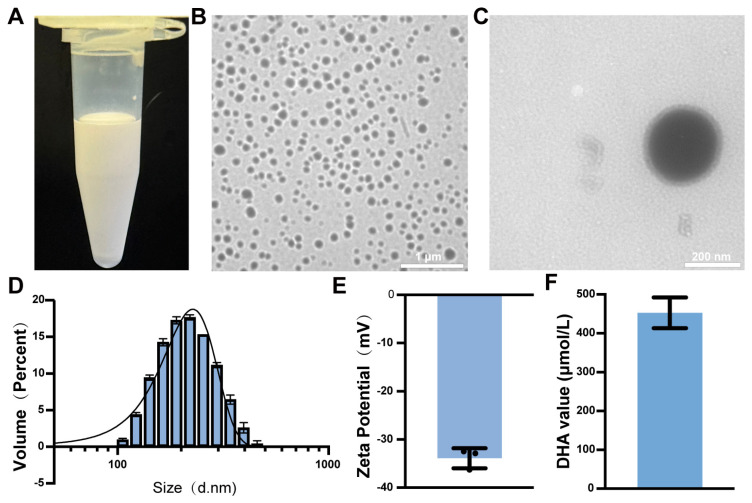
The characterization of FE. (**A**) Digital image of FE solution, (**B**,**C**) TEM images of FE at different magnifications, (**D**) FE particle size distribution, (**E**) FE zeta potential, (**F**) DHA content analysis.

FE presented as a milky white, uniform liquid (Figure 2A). Its microstructure was characterized using TEM and DLS. TEM images revealed that FE exhibited a uniform droplet morphology (Figure 2B,C). DLS analysis further indicated that the particles had a mean hydrated particle size of approximately 215 ± 11 nm and a zeta potential of −33.9 ± 2.4 mV (Figure 2D,E). A quantitative analysis of the DHA content in FE revealed a concentration of approximately 480 μmol/L (Figure 2F). These results collectively confirmed that FE possessed a homogeneous particle size distribution and stable surface electrochemical properties. Studies have indicated that negatively charged materials exhibit higher biocompatibility [36,37].

Medium/long-chain triglyceride emulsions are intravenous nutritional support agents approved by the U.S. Food and Drug Administration (FDA), primarily used to provide essential energy and fatty acids for patients unable to obtain nutrition orally or enterally. This formulation is widely used clinically to reduce postoperative infection risks and other complications, as well as mitigate parenteral nutrition-associated liver injury [38,39,40]. However, its role in bone repair has not been systematically investigated [41,42,43,44].

### 3.2. Biocompatibility Assessment

**Figure 3:**
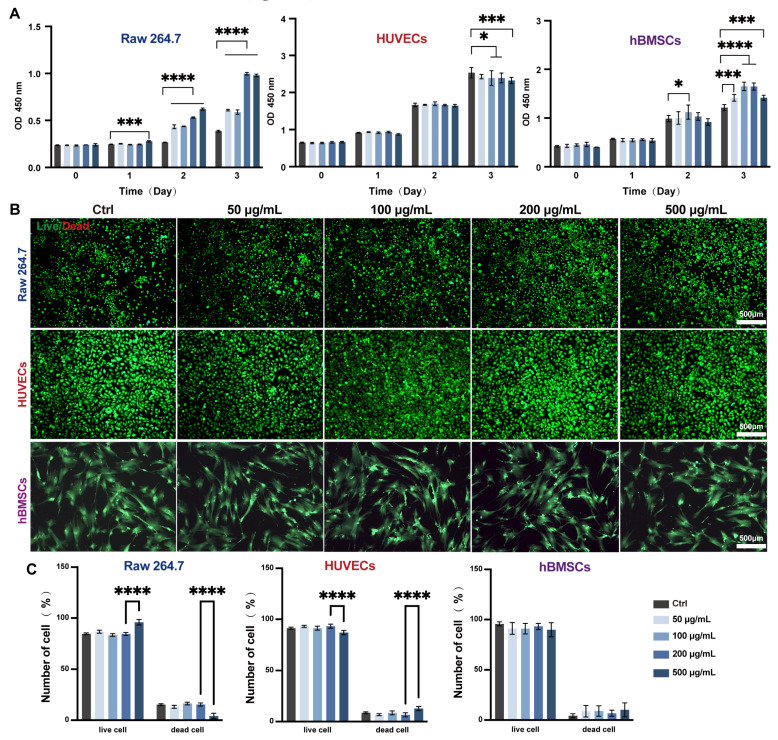
Biocompatibility assessment of ferric chloride (FE). (**A**) CCK-8 assay results; (**B**) representative live/dead cell staining images of Raw 264.7, HUVEC, and hBMSC cells treated with different concentrations of FE; and (**C**) semi-quantitative analysis of live/dead staining for the three cell types. * *p* < 0.05, *** *p* < 0.001, **** *p* < 0.0001.

To verify the biocompatibility of FE, CCK-8 assays and live/dead staining experiments were conducted on Raw 264.7 cells, HUVECs, and hBMSCs. Results indicated that FE concentrations below 200 μg/mL had no adverse effects on the survival rates of Raw 264.7 cells and hBMSCs, while exhibiting a concentration-dependent pro-proliferative effect. However, at concentrations exceeding 200 μg/mL, its proliferative effect on hBMSCs diminished. At higher concentrations, FE mildly inhibited the proliferation of HUVECs (Figure 3A). Cell viability was qualitatively assessed using a live/dead cell staining method, wherein live cells and dead cells were labeled with Calcein-AM (green fluorescence) and propidium iodide (PI, red fluorescence), respectively. Following one day of treatment with varying concentrations of FE, microscopic examination revealed normal morphology in all three cell types (Figure 3B). Further quantitative analysis indicated that the proportion of viable cells in each treatment group exceeded 90%, confirming that FE exhibited no significant cytotoxicity towards these three cell types at the tested concentrations (Figure 3C).

In summary, we observed that within non-toxic concentration ranges, FE promotes the proliferation of Raw 264.7 cells and hBMSCs, whilst exhibiting a slight inhibitory effect on HUVEC proliferation. We hypothesize that this cell type-specific response may relate to FE’s composition and metabolic differences between the cell types. As a commonly used clinical fat supplement, FE is rich in multiple unsaturated fatty acids. Upon cellular uptake, these fatty acids undergo mitochondrial β-oxidation to generate additional ATP, thereby enhancing energy metabolism. For cells such as Raw 264.7 macrophages and hBMSCs, which possess high plasticity and proliferative potential, this supplementary energy supply likely promotes proliferation and division. However, HUVECs, as endothelial cells, exhibit heightened sensitivity to lipid peroxidation and oxidative stress levels within the microenvironment during proliferation, migration, and angiogenesis. High concentrations of unsaturated fatty acids may elevate the risk of lipid peroxidation, thereby exerting a mild inhibitory effect on endothelial cells. Consequently, while FE demonstrates favorable overall biocompatibility, its finely tuned regulatory effects vary across different cell types.

Based on the aforementioned biocompatibility assessment results and considering the responses of different cell types, we ultimately selected 200 μg/mL FE as the uniform treatment concentration for all subsequent experiments. This ensures its biological functions are maximized within a safe range.

### 3.3. Anti-Inflammatory Properties of FE

**Figure 4:**
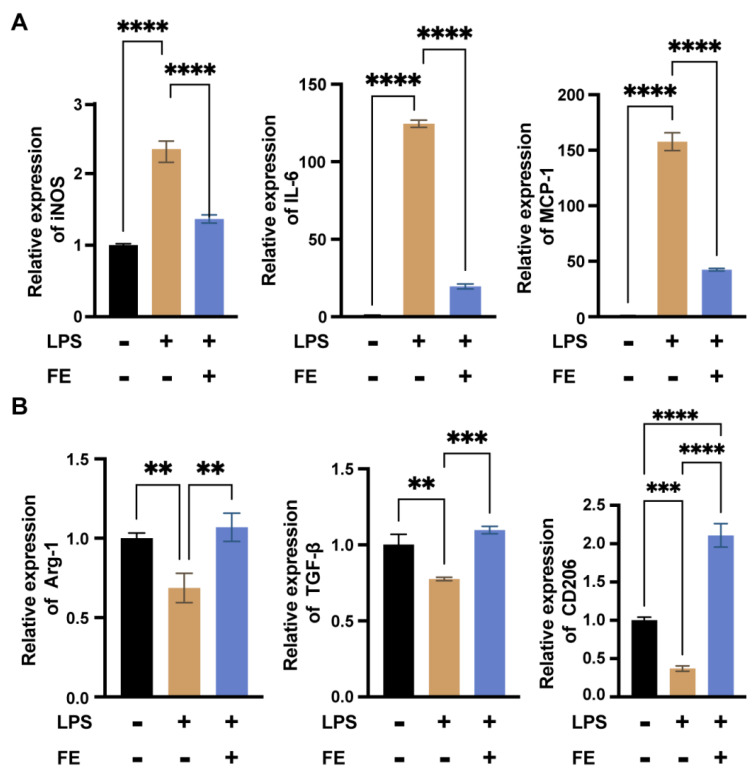
Analysis of the immunomodulatory effect of FE. (**A**) Pro-inflammatory factor mRNA expression, (**B**) anti-inflammatory factor mRNA expression. ** *p* < 0.01, *** *p* < 0.001, **** *p* < 0.0001.

To evaluate the regulatory effects of FE on the immune microenvironment, this study conducted a comprehensive investigation at both the transcriptional and protein levels. Experimental results indicated that under LPS stimulation, FE exhibited significant anti-inflammatory effects, effectively downregulating mRNA expression of the pro-inflammatory markers iNOS (1-fold), IL-6 (6-fold), and monocyte chemotactic protein-1 (MCP-1) (3-fold) (Figure 4A). FE also upregulated the mRNA expression of anti-inflammatory markers, including arginase-1 (Arg-1) (1-fold), TGF-β (1.25-fold), and CD206 (4-fold) (Figure 4B).

**Figure 5:**
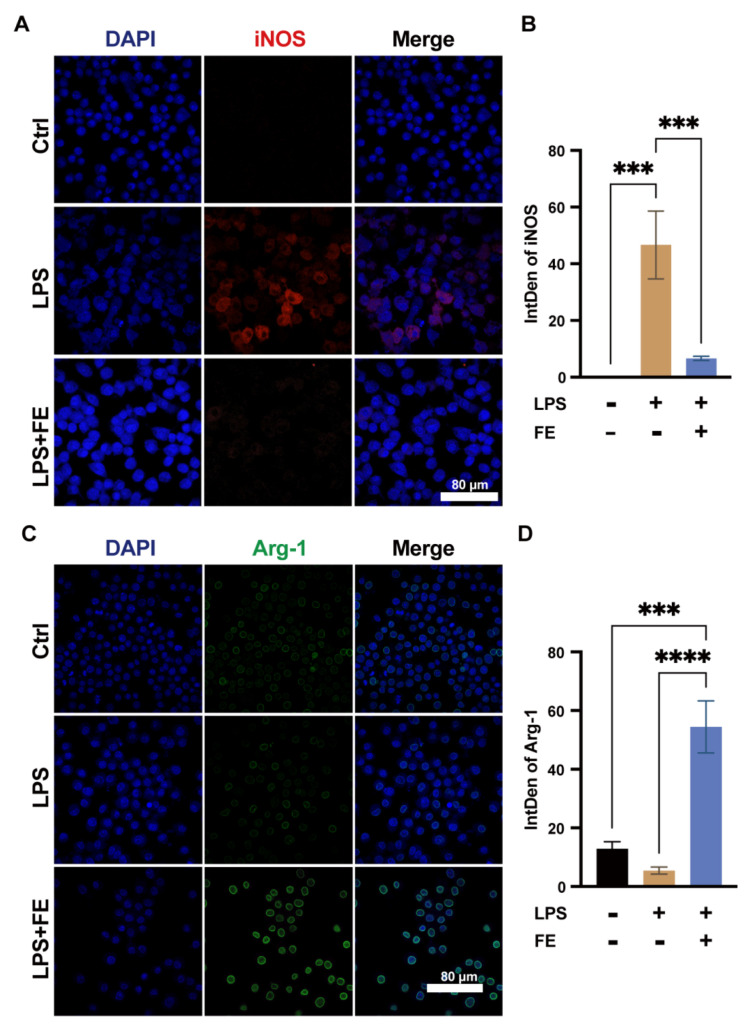
Analysis of the immunomodulatory effect of FE. (**A**,**B**) Immunofluorescence validation of the pro-inflammatory cytokine iNOS, (**C**,**D**) immunofluorescence images of the anti-inflammatory cytokine Arg-1. *** *p* < 0.001, **** *p* < 0.0001.

Immunofluorescence staining revealed that pro-inflammatory marker iNOS was significantly upregulated by 50-fold following LPS stimulation, whereas FE treatment downregulated iNOS expression by 10-fold (Figure 5A,B). Anti-inflammatory Arg-1 expression decreased by 5-fold under LPS stimulation, while FE treatment markedly increased Arg-1 expression by 12-fold (Figure 5C,D).

Genetic and protein-level analyses of M1 and M2 macrophages confirmed that FE modulated the immune microenvironment by downregulating M1 macrophages and upregulating M2 macrophages. Trauma and implant materials can induce abnormal immune responses, promoting macrophage polarization toward the pro-inflammatory phenotype (M1) [45,46]. Therefore, anti-inflammatory regulation within the bone defect microenvironment is a critical factor influencing the bone regeneration process, and bone repair materials with immunomodulatory functions hold significant potential [47]. The anti-inflammatory effects of fish oil may be mediated through multiple signaling pathways. The polyunsaturated fatty acids in fish oil can inhibit NF-κB activation by suppressing IκBα phosphorylation, thereby downregulating the expression of pro-inflammatory genes such as iNOS and IL-6 [48]. FE may also act as a natural ligand for PPARγ, activating the PPARγ signaling pathway to promote macrophage polarization towards the M2 phenotype and upregulate Arg-1 and TGF-β expression [49].

### 3.4. FE Promoted Angiogenesis via Immunomodulation

To evaluate the effect of FE on cell migration and tubule formation under improved immune conditions, migration and angiogenesis assays were performed. HUVECs were treated with conditioned medium from Raw 264.7 cells treated with LPS (RC), Raw 264.7 cells treated with LPS and FE (RFE), or standard culture medium.

**Figure 6:**
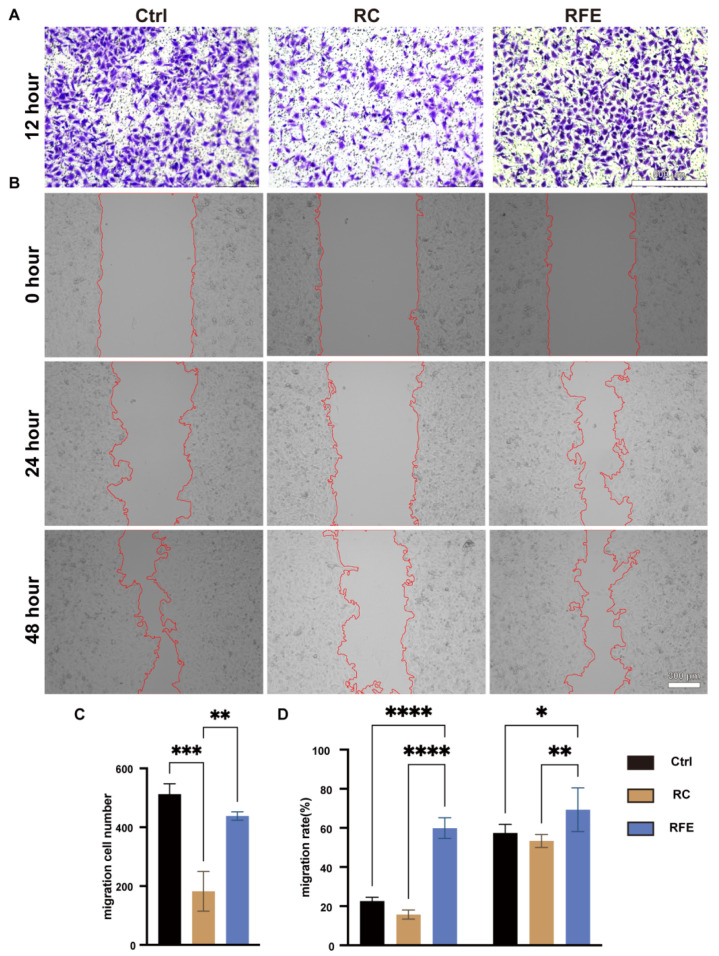
Immunomodulatory effects of FE on cell migration of HUVECs. (**A**) Transwell assay of HUVECs after being cultured in immunomodulatory angiogenesis medium for 12 h, (**B**) wound healing assay (0, 12, 24 h), (**C**) semi-quantitative analysis of Transwell results, (**D**) semi-quantitative analysis of wound healing assay. * *p* < 0.05, ** *p* < 0.01, *** *p* < 0.001, **** *p* < 0.0001.

Regarding cell migration capacity, compared with the control group, the RC group exhibited significantly reduced cell migration capacity, as evidenced by a 2.5-fold decrease in the number of cells that traversed the filter membrane in the 12 h Transwell assay. In contrast, the RFE group demonstrated markedly enhanced cell migration compared with the RC group, with a 2-fold increase in the number of cells that traversed the filter membrane in the Transwell assay at 12 h (Figure 6A,C). Additionally, in wound healing assays at 12 and 24 h, the migration rate of the RC group was lower than that of the control group. Conversely, the RFE group exhibited significantly enhanced cell migration compared with the RC group, with migration rates 3-fold higher at 12 h and 1.3-fold higher at 24 h (Figure 6B,D).

**Figure 7:**
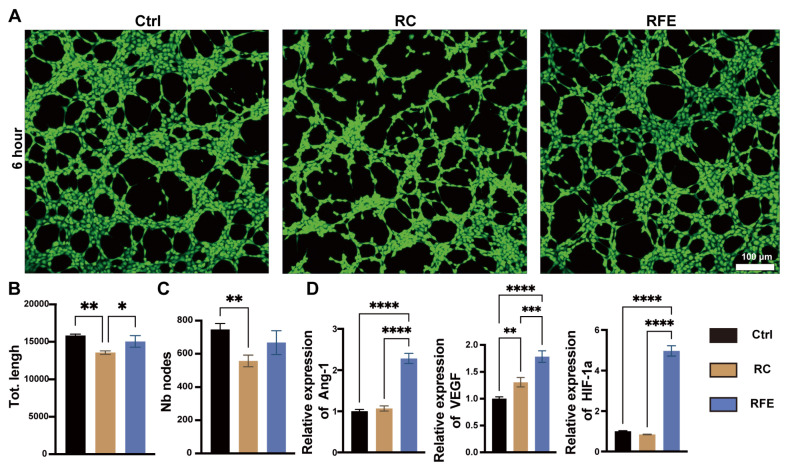
Immunomodulatory effects of FE on angiogenesis. (**A**) Angiogenesis assay after 6 h in immunomodulatory angiogenesis medium, (**B**,**C**) and their semi-quantitative analysis, (**D**) mRNA expression levels of HIF-1α, VEGF, and Ang-1 after 3 days of culture. * *p* < 0.05, ** *p* < 0.01, *** *p* < 0.001, **** *p* < 0.0001.

In the tube formation assay, the RFE group generated a more extensive and interconnected vascular network compared with the RC group. Quantitative analysis revealed significantly higher branch counts and total vessel lengths compared with the RC group (Figure 7A,C). RT-qPCR analysis indicated that HIF-1α mRNA expression was significantly upregulated approximately 4-fold in the RFE group, whereas it increased only 0.8-fold in the RC group. VEGF expression was upregulated 1.3-fold in the RC group and 1.7-fold in the RFE group. Human Angiopoietin-1 (Ang-1) expression followed a similar trend to VEGF, increasing 1.06-fold in the RC group and 2.3-fold in the RFE group (Figure 7D).

Bone, as a highly vascularized tissue, relies on the continuous delivery of oxygen and nutrients by the vascular system for the maintenance of its structure and function [50,51]. However, the inflammatory microenvironment of bone defects severely impedes angiogenesis by suppressing the release of key factors such as VEGF-A [44,50,52]. HIF-1α, as a core transcription factor in the hypoxic response, is not only induced by hypoxia but can also be activated by inflammatory signals, playing a pivotal role in linking the inflammatory microenvironment with angiogenesis. Ramanathan et al. demonstrated that lipopolysaccharide (LPS) can upregulate HIF-1α transcriptional expression in macrophages via the NF-κB signaling pathway, thereby promoting VEGF production and enabling the angiogenesis switch function of macrophages [53]. Qi et al. further confirmed that HIF-1α directly binds to the promoter region of the chemokine Ccl7, promoting macrophage recruitment through transcriptional regulation and thereby mediating vascular inflammation and remodeling. These studies reveal the key transcriptional mechanisms by which HIF-1α regulates angiogenesis under inflammatory conditions [54]. Wound healing assays and angiogenesis experiments indicated that FE enhanced angiogenesis by improving the immune microenvironment, thereby establishing foundational conditions for osteogenesis at bone defect sites.

### 3.5. Immunomodulatory Osteogenesis by FE

**Figure 8:**
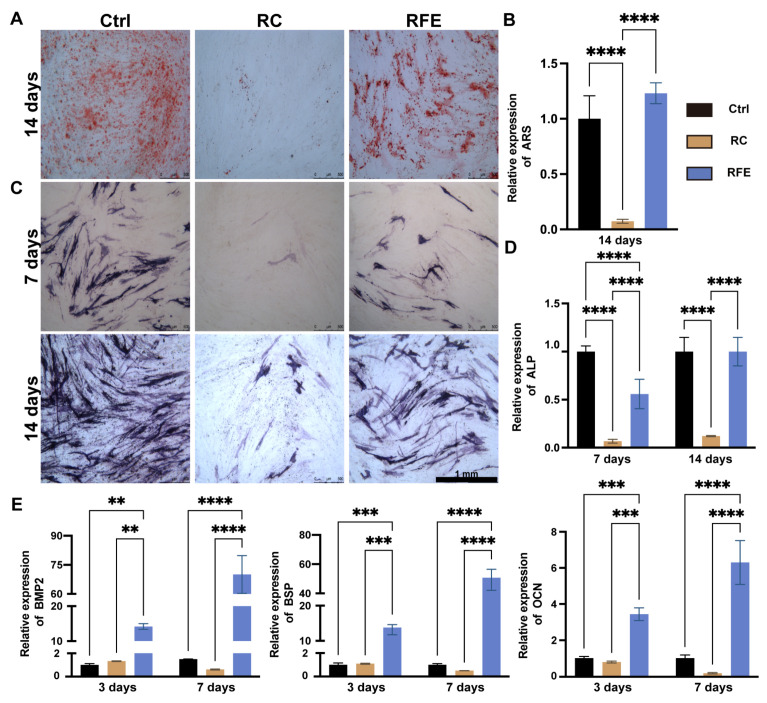
Immunomodulatory effect of FE on osteogenic differentiation of hBMSCs. (**A**,**B**) ARS staining after 14 days of culture in immunomodulatory osteogenic medium; (**C**,**D**) ALP staining after 7 and 14 days of culture; (**E**) detection of BMP2, BSP, and OCN gene expression after 3 and 7 days of culture. ** *p* < 0.01, *** *p* < 0.001, **** *p* < 0.0001.

To validate the osteogenic effects of FE in the immune microenvironment, we conducted ALP and ARS assays. ALP expression serves as a key early osteogenic marker. Results demonstrated that, at both 7 and 14 days, ALP expression was significantly suppressed in the RC group compared with the C group. However, this suppression was markedly restored in the RFE group, which exhibited significantly higher ALP levels than the RC group (Figure 8C,D). After 14 days of osteogenic induction, cell mineralization was assessed via ARS staining, with calcium deposits dissolved using 10% CPC for semi-quantitative analysis. Results showed significantly fewer calcific nodules in the RC group compared with the control group, while the RFE group exhibited markedly more red mineralized nodules than the RC group (Figure 8A,B). Additionally, we measured expression levels of osteogenesis-related genes BMP2, bone sialoprotein (BSP), and osteocalcin (OCN). Results showed that after 3 days of osteogenic induction, the RFE group exhibited approximately 14-fold higher BMP2 expression, 14.5-fold higher BSP expression, and 3.4-fold higher OCN expression compared with the control group. After 7 days, expression levels of BMP2, BSP, and OCN in the RFE group were markedly upregulated, reaching approximately 70-fold, 50-fold, and 6.3-fold, respectively, relative to the control group (Figure 8E).

Excessive inflammation inhibits osteogenic differentiation, as pro-inflammatory cytokines have been shown to impede the expression of osteogenic genes in osteoblast-like cells [55]. To overcome the barriers to bone regeneration under pathological conditions, an ideal bone repair material must effectively induce osteogenic differentiation even within an inflammatory microenvironment^12^. Experiments have confirmed that FE effectively induced osteogenic differentiation under inflammatory conditions, demonstrating its potential as a promising bone repair material.

## 4. Conclusions

To address the challenge of bone repair, immune regulation plays a crucial role to promote vascularized bone regeneration during the bone healing process. This study evaluated the effects of FE on promoting bone repair by modulating the inflammatory environment. FE is a clinically used dietary supplement, and cellular viability studies have demonstrated its excellent biocompatibility. The anti-inflammatory effects of FE may be mediated through multiple signaling pathways. Firstly, the polyunsaturated fatty acids in FE can inhibit NF-κB activation by suppressing IκBα phosphorylation, thereby downregulating the expression of pro-inflammatory genes such as iNOS and IL-6. Secondly, fatty acids act as natural ligands for PPARγ, activating the PPARγ signaling pathway to promote macrophage M2 polarization and upregulate Arg-1 and TGF-β expression. The synergistic interaction of these pathways collectively mediates FE’s immunomodulatory effects. This study further confirms that FE significantly downregulates the pro-inflammatory marker iNOS while upregulating the anti-inflammatory cytokine TGF-β, demonstrating distinct anti-inflammatory activity. Building on this, an inflammatory environment was simulated in vitro to evaluate the effects of FE on HUVECs and hBMSCs. Results showed that FE significantly promoted the secretion of VEGF and BMP-2. Under inflammatory conditions, FE enhanced endothelial tube formation, cell migration, ALP activity, and calcium nodule deposition. These findings indicate that FE can promote angiogenesis and osteogenic differentiation in vitro by modulating inflammatory responses. In summary, this study reveals the potential of FE to regulate the inflammatory microenvironment at the cellular level and enhance bone repair-related cellular functions, providing preliminary theoretical support for subsequent in vivo studies and the development of bone tissue engineering materials.

## Figures and Tables

**Table 1:** The primer sequences used for qRT-PCR.

Gene	Species	Forward Primer (5′–3′)	Reverse Primer (5′–3′)
*GAPDH*	*Mouse*	CATGTTCCAGTATGACTCCACTC	GGCCTCACCCCATTTGATGT
*iNOS*	*Mouse*	ACCTTGTTCAGCTACGCCTT	CATTCCCAAATGTGCTTGTC
*MCP-1*	*Mouse*	CACTCACCTGCTGCTACTCA	TCAGATTTACGGGTCAACTTCAC
*IL-6*	*Mouse*	CACATGTTCTCTGGGAAATCG	TTGTATCTCTGGAAGTTTCAGATTGTT
*Arg-1*	*Mouse*	CCAGAAGAATGGAAGAGTCAGTGT	GCAGATATGCAGGGAGTCACC
*TGF-β*	*Mouse*	ACTGGAGTTGTACGGCAGTG	GGGGCTGATCCCGTTGATT
*CD206*	*Mouse*	TCATCCCTGTCTCTGTTCAGC	ATGGCACTTAGAGCGTCCAC
*VEGF*	*Human*	TAGAGTACATCTTCAAGCCGTC	CTTTCTTTGGTCTGCATTCACA
*ANG-1*	*Human*	AGCGCCGAAGTCCAGAAAAC	TACTCTCACGACAGTTGCCAT
*HIF-1α*	*Human*	GAACGTCGAAAAGAAAAGTCTCG	CCTTATCAAGATGCGAACTCACA
*BMP2*	*Human*	CGAAATTCCCCGTGACCAGA	TGTTTCTCCTCCAAGTGGGC
*BSP*	*Human*	GAACCTCGTGGGGACAATTAC	ATCATAGCCATCGTAGCCTTG
*OCN*	*Human*	GCAGCGAGGTAGTGAAGAGAC	CAACTCGTCACAGTCCGGATT
*GAPDH*	*Human*	ACAACTTTGGTATCGTGGAAGG	GCCATCACGCCACAGTTTC

## Data Availability

The original contributions presented in this study are included in the article. Further inquiries can be directed to the corresponding authors.
